# Effectiveness and acceptability of two insertable device models for non-surgical management of obstetric fistula: protocol for a hybrid type I randomized crossover trial

**DOI:** 10.1186/s12905-025-03823-y

**Published:** 2025-07-04

**Authors:** Nessa Ryan, Gabriel Y. K. Ganyaglo, Joonhee Park, Tracy Kuo Lin, Joanna Pozen, Avni Mittal, Alison M. El Ayadi

**Affiliations:** 1 Restore Health, Inc, 166 Prospect Place, Brooklyn, NY 11238 USA; 2 Global Health Program, New York University School of Global Public Health, 708 Broadway, New York, NY 10003 USA; 3 Department of Obstetrics and Gynaecology, Korle Bu Teaching Hospital, Guggisberg Ave, Accra, Ghana; 4 Institute for Health & Aging, Department of Social and Behavioral Sciences, University of California, San Francisco, 490 Illinois Street, 12th floor, San Francisco, CA 94158 USA; 5 Rutgers New Jersey Medical School, 185 S Orange Ave, Newark, NJ 07103 USA; 6 Department of Obstetrics, Gynecology and Reproductive Sciences, University of California, San Francisco, 550 16th Street, Third Floor, San Francisco, CA 94158 USA

**Keywords:** Vesicovaginal fistula, Incontinence management, Insertable device, Effectiveness, Acceptability, Stigma, Quality of life

## Abstract

**Background:**

Obstetric fistula is a traumatic and stigmatized maternal morbidity often resulting in severe urinary and fecal incontinence. Women with fistula face multi-level barriers to surgical repair culminating in delays. Unfortunately, no acceptable temporizing measures to contain incontinence of urine exists. An insertable vaginal cup, alone or connected to a leg bag, has potential for improving incontinence management for women awaiting surgery or those whom surgery was unsuccessful, but effectiveness and acceptability are unknown.

**Methods:**

We describe a four-year clinical trial and nested qualitative study to examine the effectiveness and acceptability of an insertable vaginal cup to manage fistula urinary incontinence and understand fistula management costs. Two intervention models will be compared to a control: (1) vaginal cup (‘cup’), and (2) vaginal cup attached via tubing to a leg-secured urine collection bag (‘cup+’). Using a cross-over design, up to 100 participants will be block randomized to one of two sequences of leaking freely (no intervention), cup, and cup + at four fistula centers in Ghana and Kenya and observed for four days (400 total observations). Data will be captured through interviewer-administered survey, clinical exam and checklist. After clinic-based assessment, participants are individually randomized for cup or cup + for home use for up to 3 months and surveyed monthly. Effectiveness will be evaluated through quantitative comparison of urinary leakage (6 h and 24 h) and patient-reported quality of life (1–3 months) between cup, cup+, and leaking freely. Acceptability will be assessed quantitatively (1–3 months) and via in-depth interview among selected trial participants (*n* ~ 30) and potential implementers (*n* ~ 20).

**Discussion:**

This implementation study will inform the effectiveness and acceptability of the cup and cup + interventions as temporizing management strategies for fistula urinary incontinence. If the cup/cup + is effective and acceptable, this study will provide insight for future trials and cost-effective assessments in settings where fistula is prevalent. Expanding the evidence base on non-surgical temporizing management options will inform comprehensive fistula care through tertiary prevention and is likely to reduce vulnerability to stigma and improve economic opportunity and quality of life.

**Trial registrations and dates:**

ClinicalTrials.gov NCT05444504 (Date of registration: July 6, 2022). Pan African Clinical Trial Registry 202,209,466,217,416 (Date of registration: 9/22/2022). Ghana FDA Certificate FDA/CT/231 (Date of approval: 3/30/2023).

## Background

Prevalence estimates suggest that more than 500,000 women and girls live with the birth injury obstetric fistula, primarily in lower-resource settings [1, 2] While fistula repair capacity is increasing globally, [3–5] with as many as 100,000 annual incident cases,^1^, ^2^ current surgical capacity is inadequate and many women face surgical treatment delays [6–8]. For example, in Ghana, where fistula incidence is estimated at ~ 1 per 1000 births, [9] impacting ~ 1,350 women annually, annual repair capacity is < 150; thus, Ghanaian women report living with fistula for a median of eight years prior to repair (Interquartile Range (IQR): 4–14) [10]. Similar substantial care delays are observed in Kenya, where women have been reported to wait on average 8.1 years (SD 9.2) for surgery. The substantial and persistent discrepancy between surgical capacity and new fistula cases in Ghana, Kenya, and in other countries where fistula is prevalent leaves a significant backlog of women in need of care and facing multi-level barriers to timely access to surgical fistula management.

Chronic urine leakage from obstetric fistula results in stigma, including discrimination, social isolation, and shame causing mental health and economic consequences [11–14] that significantly reduce quality of life [15–21]. Women with fistula cope with urine leakage with low-cost materials, however, with challenges [22–25]. In many settings where obstetric fistula occurs, diapers or sanitary pads are prohibitively expensive or unavailable; thus women use absorptive fabrics that hold urine against the skin, causing severe irritation, and require frequent washing [26]. Effective options for self-management of fistula leakage are inaccessible for most. Thus, increasing accessibility to safe, low-cost tools are needed, including acceptable, non-surgical innovations to manage fistula urinary incontinence until timely access to surgical fistula repair is assured.

Insertable devices, or technologies that reside within the body, have many applications to improve women’s health and quality of life [27, 28]. An insertable vaginal cup is an acceptable, safe, and effective solution for preventing leakage of menstrual blood and eliminating odor among women and girls worldwide including settings where fistula is prevalent [29–33]. Incidental findings in case reports suggest the utility of the menstrual cup for control of urine leakage in women with fistula [34–36] however, no systematic evaluation of the effectiveness of the cup within a fistula-endemic setting has been conducted. The cup is durable, reusable, and low-cost in addition to being concealable to prevent additional stigma burden. It sits low in the vagina, and thus is suitable for collecting leakage for selected vesicovaginal fistulas (VVF). Urine is emptied from the cup after removal or via valve release while inserted.

Our collaborative team of physicians and researchers from University of Ghana and New York University were the first to systematically evaluate efficacy and acceptability of an insertablevaginal cup for short-term vesicovaginal fistula management at a health facility in Ghana within a feasibility study [37, 38]. The mean leakage reduction with cup use over two hours was 61% (95% CI: 35.9–86.2), a difference users perceived in reduced wetness (10/11), including a 64% mean leakage reduction among the five participants with previous surgical attempts. Acceptability was high; women could easily insert (8/11), remove (8/11), and comfortably wear (11/11) the cup, and most (10/11) would recommend it. No adverse effects attributable to use of the cup were observed on gynecological exam. The data collection tools (e.g., pad test and questionnaire on perceived reduction of leakage and acceptability) were appropriate and easily implemented in the local context. These findings suggested robust evaluation of short and long-term effectiveness is warranted along with continued evaluation of user acceptability, including how use of the cup could supplement or replace existing coping strategies [4, 39, 40].

Results of the feasibility study suggested two key design modifications to target an insertable device for fistula management [41]. A valve would allow the user to empty the cup without removing it from the vagina, thus reducing the emptying frequency (Table 1). Results also suggested that users may benefit from greater storage capacity through connecting the valved cup via rubber tubing to a drainage bag attached to the leg by soft elastic bands and concealed under clothing. The larger capacity of the drainage bag (540 ml) could allow users to empty the urine less frequently compared to the cup alone (35 ml) without worrying about leakage. This would be beneficial when women lack immediate access to water and a safe, private space to empty and wash their cup. Women coping with fistula already maintain strict hygiene practices, thus additional cleaning and hygiene needs are not concerning [42, 43]. The cup alone may be more appropriate for shorter duration or among women with less severe leakage while cup plus leg bag (cup+) may be more appropriate for longer duration (e.g., traveling greater distance without latrine access) or among women with more severe leakage. Acceptability could vary by model, change over time and is dependent on multiple factors and contexts.

**Table 1:**
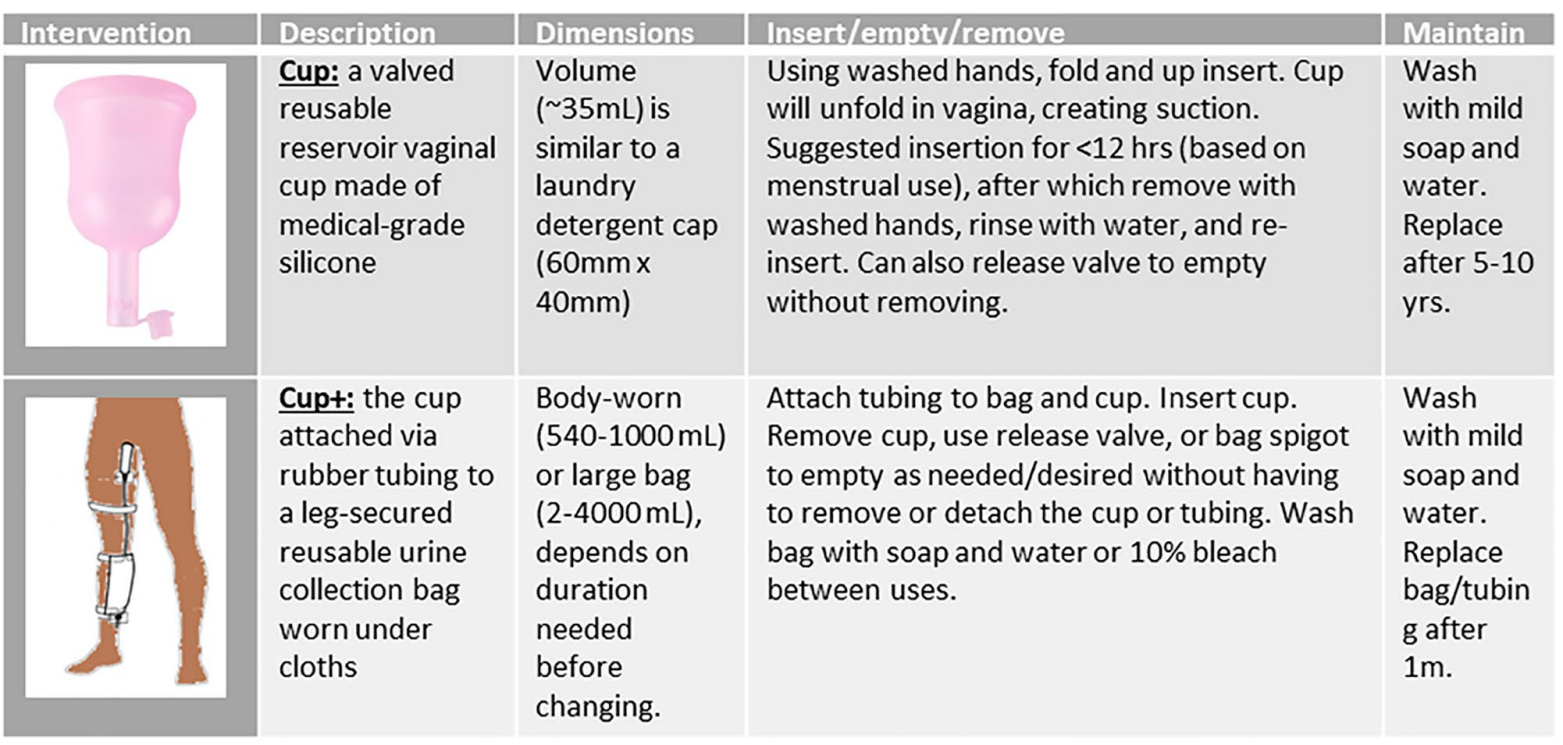
Description of the cup and cup + Intervention models

Cost is an important implementation outcome for this and any fistula management intervention. Although qualitative studies have suggested women devote significant time and effort to non-surgical fistula management, [14, 26, 44, 45] no rigorous quantification of material and opportunity costs exists. As such, the economic burden of fistula is not well understood, which limits valid estimation of the benefits of fistula interventions. Micro-costing of non-surgical management is needed to quantify the burden of managing fistula urine leakage to comprehensively characterize the social and economic impact of any fistula management intervention. Cost-effectiveness of fistula surgery has been previously evaluated in Uganda, showing that for a hypothetical 20-year-old woman with fistula, surgery was estimated to decrease the lifetime disability burden from 8.53 DALYs to 1.51 DALYs, yielding a cost of $54 per DALY averted [46]. However, cost comparisons and cost-effectiveness analysis have yet to be conducted between methods of self-management of fistula. Various aspects contribute to financial and economic cost of fistula management; 47] valved menstrual cups that would meet the needs identified in our prior research are now available (cost of $4.50 per unit) and tubings with leg bags are readily available (combined cost of $6.10 per unit).

As acceptability, appropriateness, and other implementation barriers have been previously identified when implementing evidence-based tools for incontinence management, [48, 49] menstrual hygiene management, [31] and surgical management of fistula, [50, 51] contextualizing implementation while evaluating the impact of the cup and cup + is critical. Therefore, this study will evaluate the effectiveness and acceptability of a valved vaginal cup and a valved vaginal cup with tubing for non-surgical management of VVF, estimate fistula-related management costs, and understand the implementation context for the cups. Importantly, the comparison for effectiveness outcomes is not surgery but free leakage. Identification of effectiveness and acceptability for either intervention model could influence a shift in clinical fistula management practice paradigms. Qualitative findings from community assessment of the cup and cup + among users and fistula stakeholder interviews will help explain quantitative results regarding efficacy, effectiveness, or adherence.

We hypothesize that limits to the cup/cup + effectiveness for fistula incontinence management are likely to be attributable to acceptability and adherence [52]. While the use of cup/cup + for fistula incontinence management is novel, cups and other intra-vaginal devices for menstrual management, [53 contraception,^54^] and prevention of sexually transmitted infection [55, 56] are used when available and accessible and have been found to be acceptable throughout sub-Saharan Africa [57] To support a multi-dimensional understanding of both user and implementer acceptability and cost of cup/cup+, use of behavioral frameworks like the Theoretical Framework of Acceptability [58] can help identify how acceptability shapes user adherence and uptake and guide identification of optimal user and implementer characteristics. Our theory-oriented approach will inform generalizability of our effectiveness findings, provide an understanding of context, and help identify implementation strategies that may improve implementation outcomes, such as acceptability, appropriateness, and cost.

## Methods

### Study design

**Fig. 1:**
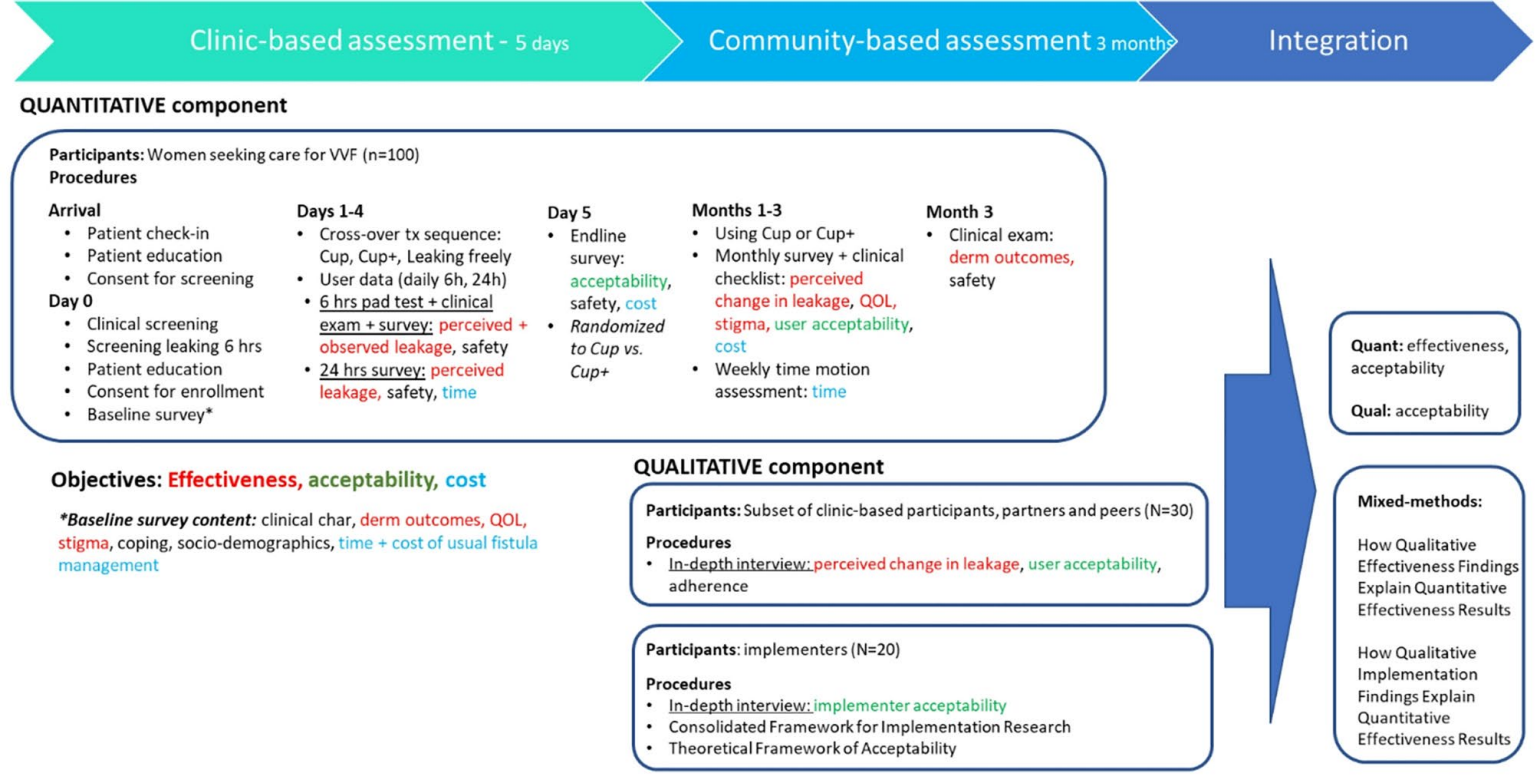
Mixed-methods study diagram

This hybrid type I randomized crossover phase III clinical trial and nested qualitative [59] study employing a concurrent mixed-methods approach (Fig. 1) [60] seeks to longitudinally quantify the effectiveness and comparative effectiveness of the cup and cup + to manage vesicovaginal fistula urinary incontinence (Aim 1) among up to 100 women in Ghana and Kenya who have not yet accessed treatment or have failed surgical repair, examine acceptability of the cup and cup+ (Aim 2), and estimate the material and opportunity costs of non-surgical fistula management (Aim 3).

**Fig. 2:**
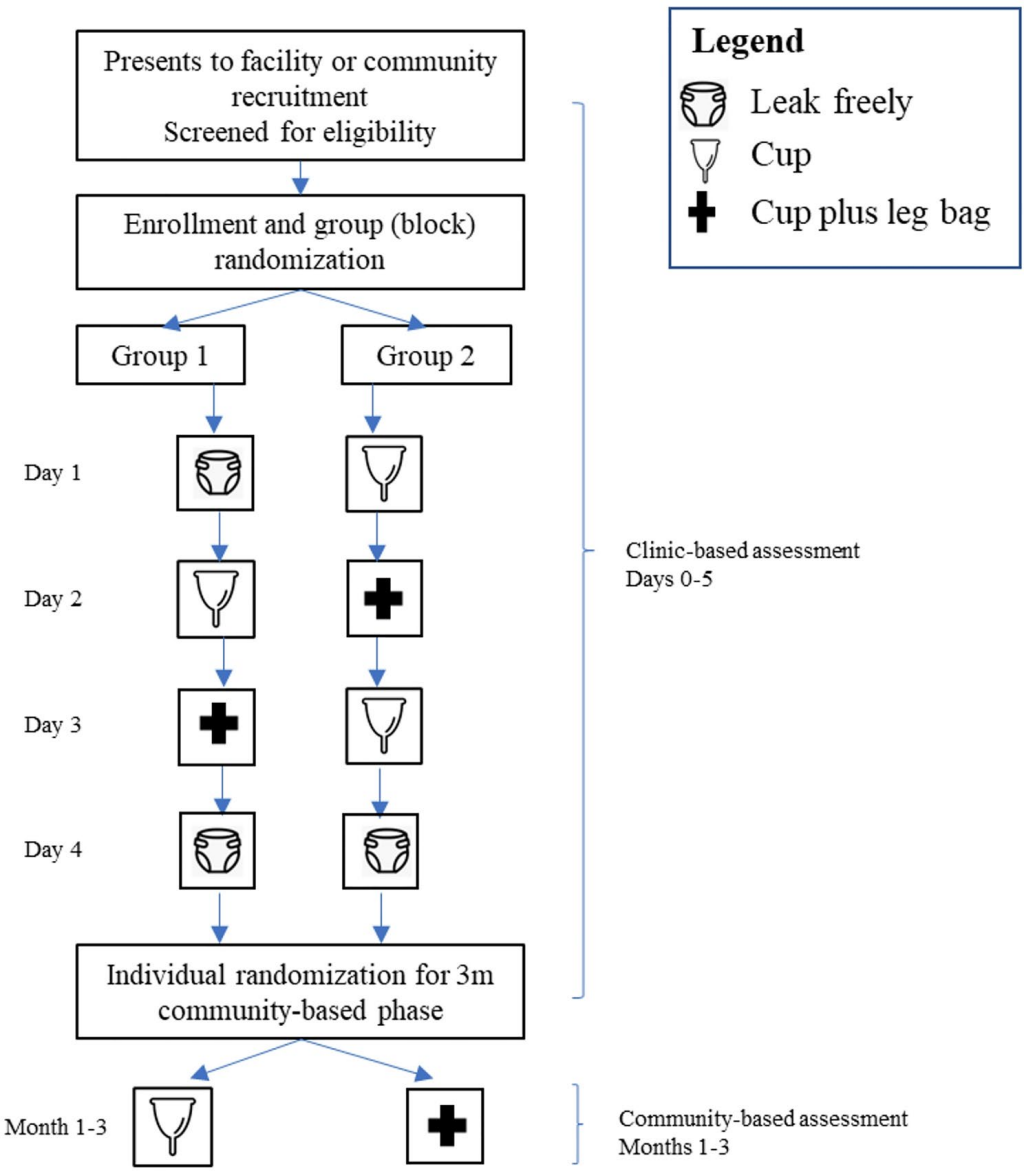
Participant flow diagram across clinic and community-based study components

At the health facility, participants will first undergo baseline assessment of leaking freely (six hours). Participants will be block randomized within a crossover design [61] to varying sequences of leaking freely, using the cup, and using the cup+ (Fig. 2). During clinic-based assessment, the study will quantitatively evaluate short-term comparative efficacy (at six hours) between the cup and the cup + and assess medium-term comparative effectiveness (at 24 h). Participants will be subsequently individually randomized to take home either the cup or the cup + as part of a community assessment of longer-term effectiveness, including change in quality of life (after one to three months). Preliminary clinic- and community-based assessment results will connect the qualitative phase by informing the qualitative stakeholder interview guide and purposive selection of a sub-sample of purposively selected trial participants, partners, and peers (*n* ~ 30). These individuals will be interviewed for an in-depth understanding of user-cup interactions, acceptability, stigma, and quality of life. We will also begin to qualitatively examine the implementation context for the cup and the cup + as perceived by providers (*n* ~ 20).

This mixed-methods design allows for a more complete understanding of non-surgical management than quantitative or qualitative approaches alone, [62] emphasizing our primary outcome of effectiveness via robust assessment of clinical and patient-reported outcomes, and providing important acceptability and cost burden data to inform future implementation. Our approach maintains a focus on rapid translational gains for clinical intervention and useful information for key decision-makers.

### Study setting

Participants will be recruited from four facilities in Ghana and Kenya. In Ghana, the study will enroll participants at Mercy Women’s Catholic Hospital in Mankessim and Tamale Fistula Centre at Tamale Central Hospital in Tamale. In Kenya, the study will enroll participants at the Gynocare Women’s and Fistula Hospital in Eldoret and WADADIA-Habibat Mother and Child Holistic Health and Training Hospital in Malindi.

### Stakeholder engagement

Trial implementation and interpretation will be supported by a Community Advisory Board and the Ghana and Kenya National Fistula Task Forces. The Community Advisory Board (CAB; *n* = 5–8) will provide input on recruitment, study procedures, findings, and interpretation to increase community engagement and improve research quality. An effort will be made to include board members who are involved in clinical operations and can provide some institutional perspective. Other CAB members may represent social organizations where this intervention could be implemented, such as community-based organizations with fistula programming. Others will represent strategic regional and national fistula organizations.

### Study population and sample size

#### Quantitative

We will recruit up to 100 women with VVF to participate in the quantitative study activities (Aims 1–3). Study participation will not delay time to surgery for study participants through careful planning of recruitment efforts in alignment with timelines of surgical availability and through early study exit from community-based activities if surgery becomes available sooner. Our sample size calculation is not dependent on balance between study sites, thus the breakdown of participants by study site is not pre-defined but will depend on recruitment activities. Detailed inclusion and exclusion criteria are as follows:

- *Inclusion criteria*: VVF confirmed by dye test and clinical exam at least three centimeters (cm) from the external urethral orifice regardless of size, adequate vaginal capacity to accommodate the cup (per physician), willing to insert and remove cup/cup+, clear understanding of the study procedures and willing to participate fully, not yet undergone surgical fistula repair or previously failed surgical repair, at least 6mo post-surgery if previous fistula repair, ≥3months post-delivery if recent birth, age 18 + or emancipated minor per Ghanaian or Kenyan law, and ability to speak English or local language.
- *Exclusion criteria*: Presence of any rectovaginal fistula (i.e., alone or in combination with VVF); not able to provide informed consent (e.g., incapacitated); does not leak more than 6 ml over 6 h during screening examination; and fistulas deemed curable via prolonged catheterization. Pregnant individuals will not be excluded from the study participation if they meet the inclusion criteria specified above. Pregnant or menstruating women will not be excluded and their experiences and outcomes will be compared to the larger sample.

Cross-over trial designs provide greater efficiency than parallel designs due to within-subject treatment comparisons where each subject serves as their own control, resulting in lower sample size requirements for statistical power. Our sample size of up to 100 participants (400 observations) is calculated to assess: (1) a ≥ 65% reduction at six hours in observed urinary leakage, and (2) a mean 40% quality of life difference at three months between the cup and cup+ (measure range 0-100; Supplementary Figures). We expect > 80% retention over three months study follow-up based on our research with 12-months follow-up with 98% retention with this target population in Uganda^63^ and 3-months follow-up in Ghana with 84% retention, [64, 65] and have inflated our target sample size conservatively.

#### Observed leakage

Minimum difference was estimated from our efficacy study plus expert clinical opinion as the minimum clinically significant leakage reduction from pad test [24]. Analytical sample size was calculated at 50 using an ANCOVA model with a 2-tailed, alpha error = 0.025 to obtain statistical power of 0.8–0.9.

#### Quality of life

Using dependent t-test with 2-tailed, alpha error = 0.05, estimated mean difference of effect between the cup and cup + of 40%, and power 0.85 to avoid type 1 error, required sample size is 59. Estimates of the effect are based on data collected within a longitudinal study of post-surgical fistula repair patients in Uganda, [66] wherein participants were asked about quality of life using the post-repair fistula reintegration measure which is standardized to a range of 0-100 [67]. Participants reported a mean of 36.7 (SD = 19.0) at the time of fistula repair (referring to living with fistula) and a mean of 78.0 (SD = 22.7) at follow up (6 months post-repair), reflecting a mean change of 40.5 (SD 28.1): 54.2 (SD 14.5) among those who were not leaking urine and 13.1 (SD 28.9) among those who continued leaking urine. The cup + is estimated to have a comparable improvement on quality of life as surgical repair, while the cup, based on our observations during our feasibility study [37] and on expert opinion, is estimated to have a smaller effect on quality of life over the long-term (3 months) as it has 1/15th of the capacity for urine storage and thus will need more frequent emptying than the cup+.

#### Qualitative

Study staff will recruit ~ 30 trial participants, partners, and peers and ~ 20 providers (obstetrician-gynecologists, midwives/nurses, traditional birth attendants, community health workers). Participants will be targeted based on criterion purposive sampling to identify a diverse sample of those with high or low acceptability; high, low, or no subjective effectiveness, and severe or not severe stigma experiences. Each invited trial participant will be encouraged to invite one to two partners or peers to provide additional context. Final sample size will be determined by iterative appraisal of theme saturation as data are collected, [68] although a priori estimations suggest 20 to 30 participants will be sufficient. Interviews will seek to understand communication with partners and peers regarding fistula management decision making, context for fistula management decision making, adherence, cost, relationship dynamics, social support, and caregiver or courtesy stigma.

### Participant recruitment

#### Quantitative

Participants will be recruited from women accessing fistula repair services at study-engaged sites and through community outreach by trained nurses. Potential participants will undergo a two-stage informed consent process with trained study staff for communication of screening procedures, eligibility assessment and full study procedures. Women consenting to screening will undergo preliminary clinical examination by the site physician, a fistula surgeon, to determine eligibility. Potential participants will be informed that they will receive the treatment assignments in varying order across their clinic stay.

Eligible women will be guided through the consent process, invited to participate in the study, and enrolled if they agree. Participants receive up to ~$135 USD in Ghana and ~$100 in Kenya for the six days of screening, clinic use and observation, and home use, reflecting local clinic (travel time, lost wages during facility stay, expenses for childcare, and communications) and community-based (time and effort) costs. The total participation reimbursement each individual will receive is based on their sequential engagement in study activities (i.e., clinic-based, community-based, and in-depth interview), and has been carefully estimated based on local costs and approved by the local IRBs. Participant transportation to the clinic and clinic-based expenses (e.g., food, water, bedding) are provided by the study.

#### Qualitative

*Trial participants*,* partners*,* and peers*: A sub-sample of trial participants selected for variability in reported short-term subjective and objective effectiveness and quantitative acceptability will be invited for an in-depth interview on user experience (i.e., acceptability, stigma, and quality of life) after clinic-based and/or community-based usage. This purposive and criterion sampling will allow us to examine clinical significance (i.e., what do users perceive is a meaningful amount or percentage reduction in leakage with use of cup/cup+) and identify who might be an optimal user of the cup/cup+ (including based on anatomical characteristics). In addition, and as appropriate, we will ask each recruited participant if we can talk to a male partner, another family member, or a peer to ask related questions about acceptability, as male engagement and family member support could shape interaction with women’s health services [69–71] and their decision to use the cup/cup+. *Providers*: Diverse implementers will be recruited for in-depth interview on implementer acceptability, which will inform us on how best to identify and address potential implementation challenges, beyond user characteristics. Qualitative participants may receive an additional ~$5-15 USD.

### Clinical trial procedures

#### Clinic-based procedures

After consent and enrollment of a participant, as well as baseline measurement of leakage, clusters of approximately 4 to 10 participants enrolled around the same time will be block randomized using REDCap software and the participant clusters will be allocated to one of two groups (Fig. 2). Women who are menstruating at the time of baseline measurement or expect to begin menstruation during the week of initial data collection will be noted as such and expected additional volume of blood leakage will be accounted for during data analysis to reduce measurement error.

##### Randomization

Participants will be group randomized to one of two groups of varying sequences of 24 h of either leaking freely, cup, or cup+ (Fig. 1). This crossover design uses participants as their own controls, reduces the risk of confounding due to intervention order, and captures two estimates of leaking freely (group 1) and cup (group 2) to assess any sequential differences in leaking variability. Based on the intervention mechanism of effect and lack of carry-over effect, no washout period is required between sequences. As the clinic-based component of the trial will be implemented in small groups prioritizing feasibility and quality control, randomization at the group level is preferred and will be implemented by the study data manager at the time each cohort is recruited using coin toss. The study physician and nurse will enroll participants and inform them of the treatment group for immediate participation.

#### Participant education

Once enrolled, participants will receive education, counseling, and practice on insertion, use, removal, and hygiene practices of the vaginal cup and cup with tubing per group sequence [72]. The study nurse will teach subjects how to place and remove the cup from the vagina and wear the cup with tubing connected to a collecting bag. Culturally-appropriate pelvic models will be used for education, demonstration and review. Study staff will instruct women to remove and reinsert the cup as they need or desire, supply detailed instructions about when and how to seek medical advice or help, and address participant questions [73]. Participants will also be taught how to wash the cup using soap and water and the leg bag and tubing using water and vinegar or bleach. The cup can be air dried or wiped dry. Participants will have ample opportunity to practice insertion, removal, and washing of cup by themselves, as well as the attachment, removal, emptying, and washing of the tubing and collecting bag. The importance of hand hygiene will be emphasized [72]. Study staff will explain that women should feel free to remove and reinsert the cup as they feel is appropriate. If they think the cup is getting full, which they might suspect if starting to leak, they will be encouraged to empty the cup using the valve or via removing the cup, empty and re-insert. Participants will be encouraged to ask questions during this discussion and can access supportive assistance from the study nurses. Participants will be counselled to drink enough water to allow the free flow of colorless, odorless urine [73]. All instruction and data collection will occur in the participant’s preferred local language. Given expected literacy constraints, education and training will emphasize verbal and visual strategies. Still, brief written and pictorial materials will be offered to participants for their ongoing reference, with sensitivity to protecting women’s fistula concealment, their use of the cup/cup+, and their participation in the trial.

##### Data collection

Data will be collected within the clinic-based phase via questionnaire, clinical exam (by a physician), clinical checklist (by nurse/midwife), pad test, and observation (Fig. 1; Table 2). At baseline, a gynecological exam will be carried out as potential participants are being screened and, once enrolled, the baseline questionnaire will be administered. Data will be directly entered into the REDCap mobile app to facilitate data privacy and quality. Next, all participants will be provided disposable pre-weighed adult pads or diapers for the first six hours of each treatment assignment while carrying out typical activities daily living activities on the ward. Six hours was deemed to be a clinically significant amount of time to provide women some relief from their leaking to allow them to engage in social activities or sleep uninterrupted. Additionally, six hours will likely provide those in the cup + assignment the opportunity to fill and then empty their leg bag. Lastly, six hours was deemed logistically feasible for data collectors (i.e., at the start and end of their shift). When assigned to cup or cup+, participants will also insert and wear the respective cups. Women will be encouraged to void ‘normally’ if they sense urgency and instructed to change their diapers as frequently as they see fit when they feel dampness and to deposit the used diapers into a personal plastic Ziplock bag that is then sealed until study staff can record the weight of the wet diapers. At the end of the first six hours of each treatment assignment, the weight of the participant’s assigned dry diapers will be subtracted from that of the wet diapers to calculate the volume of urine leaked, and participants will be asked questions regarding perceived effectiveness. Participants will continue with their assigned treatment until 24 h of use, at which time another questionnaire and a clinical checklist to note any adverse events will be administered by the study physician or nurse/midwife.

**Table 2:** Quantitative study measurements, data sources, and timing of assessment

Category	Measure	Data Source^a^	Clinic				Community		
			0h	6h	24h	4d	1m	2m	3m
Outcome variables									
Quality of life ^b,c^	Quality of life (Post-fistula repair reintegration instrument)^*92*^	PS	●				●	●	●
Urine leaked (observed) ^c^	Pad weight; number of pads used, food and fluid intake, voiding	PT, TM		●					
Urine leaked (perceived) ^c^	Perceived difference in leakage (cup, cup+, leak free)^90,91^	PS		●	●		●	●	●
Acceptability (patient, family, clinician) ^c^	Ease, comfort, interference, likelihood to use or recommend	PS		●			●	●	●
	Barriers to use, concerns, use decision-making process	PS				●			
**Covariates**									
*Patient-related*									
Socio-demographics ^c^	Age, education, socio-economic status	PS	●						
Obstetric history	Parity, delivery type, timing of fistula, repair attempts	PS	●						
Reproductive health ^c^	Menstruation, pregnancy status	PS	●		●		●	●	●
Other health/functional ^b,c^	Stigma ^*93*^	PS	●				●	●	●
*Fistula-related*									
Fistula characteristics ^b,c^	Fistula classification, distance from external urethral orifice	CE	●	●					
Physical signs and safety ^c^	Vaginal mucosa, vulvar dermatological, fistula etiology	CC		●	●	●			●
Physical symptoms ^c^	Fistula-related symptoms (incontinence severity, pain, mobility)	PS	●				●	●	●
Fistula management practices, time, costs ^c,d^	Time and methods used for managing leakage, pain; cost of usual odor or leakage management,^94^ participation in social activities	PS	●		●		●	●	●

Note: ^a^Data Sources include clinical checklist (CC), clinical exam (CE), patient survey (PS), pad test (PT), and time motion (TM). ^b^Measures demonstrated to be valid in this population. ^c^Measures previously used by the research team in this population. ^d^Individual cost data will include time/opportunity costs to visit clinic, payments for management time, equipment, and opportunity costs of family/ friend caretakers

### Community-based procedures

On the last clinic day, participants will be individually randomized to use of cup or cup + at home for up to three months (see Randomization; Fig. 1) for community-based assessment of the longer-term effectiveness, acceptability of the cup and cup+, and material and opportunity costs to non-surgical fistula management. We will assess perceived change in leakage associated with cup/cup + use, quality of life, acceptability and adherence via survey and clinical checklist administered at one and two months either over the phone as we have done in prior studies [63, 74] or in-person per participant and data collector preference, and semi-structured interview and clinical exam in-person at three months. Participants will be provided with airtime to ensure their ability to contact study personnel.

### Study termination

Participants will be free to discontinue use of any individual intervention assignment or withdraw from the trial completely at any point throughout the study. Criteria for study-initiated discontinuation include user perception of and/or clinical observation of adverse events such as significant pain. Study participation will not delay participant access to fistula surgery; if active study participants are called for fistula surgery, they will complete research activities through the time of surgery at which point they will be withdrawn. Outcomes will be recorded for those who discontinue or deviate from the intervention protocol, including reason for discontinuation or deviation and length of use. Participants will receive a clinical exam at the time of discontinuation or deviation.

### Qualitative study procedures

Qualitative assessment among a purposively selected sub-sample of trial participants, their partners, and peers (*n* = 30) will provide a more in-depth understanding of user experience, including acceptability, stigma, and quality of life. Interviews will be informed by the Consolidated Framework for Implementation Research (CFIR) and the Theoretical Framework of Acceptability (TFA), [58, 75] and the interview guide will be further adapted during the clinic-based assessment phase and by the community advisory board. The interview will occur at a mutually agreed-upon location for participant and researcher where the researcher can maintain appropriate privacy for the participant. Interviews will be audio recorded with permission. For trial participants, this may be at the clinic during their final study follow-up visit at up to three months or at their home, as per their preference. For implementers, this may be at their place of work or another convenient location. Interviewees will be remunerated for their time.

The acceptability survey captures Theoretical Framework of Acceptability constructs relevant for user acceptability: attitude, burden, perceived effectiveness, ethicality, intervention coherence, opportunity costs, and self-efficacy (Table 2) [58]. Interview questions will include sensitizing concepts from the five CFIR domains given their potential impact on evidence-based intervention implementation [75]. Most relevant domains for trial participants may include: intervention itself (intervention source, relative advantage, adaptability, trialability, cost, complexity) and outer setting for implementation (patient needs and resources). Most relevant domains for implementers may include: individuals involved in implementation (knowledge and beliefs about the intervention, self-efficacy), the inner and outer setting (structural characteristics, external policies, culture, networks, communications), and the process by which implementation occurs (planning, engaging, executing, reflecting, evaluating). We will also explore recently proposed adaptations to the CFIR to increase compatibility of use for lower-resource settings including to account for systems-level determinants operating independently of the implementing organization and 11 novel constructs augmenting the utility and comprehensiveness [76]. This stakeholder group will also engage in creating a list of potential implementation strategies to improve fit of implementation for this context.

### Costing study

Using time motion study (TM)^77^ and micro-costing methods, we will longitudinally survey all trial participants (*n* = up to 100) while in the facility and during community-based follow-up on fistula management practices to estimate direct costs (medical and nonmedical) and indirect costs (productivity) from the patient perspective. We will compare fistula management costs between cup, cup+, and usual non-surgical methods to inform the social impact of fistula management and future cost-effectiveness assessment. While it is often recommended that time motion is conducted with an external observer, [77] the sensitivity and stigma surrounding non-surgical fistula management makes the employment of an external observer of personal hygiene behavior inappropriate. Nevertheless, it is critical to capture individual indirect cost of non-surgical fistula management and how the cost may vary across interventions thus we rely on participant self-report of non-surgical fistula management in the community. Potential for bias will be minimized through use of standard questions focused on 24-hr recall. During community-based follow-up by the research assistants, multiple assessment points will allow us to observe change over time as well as understand the influence of community-level factors (e.g., distance to latrine, accessibility of water) on non-surgical management.

Cost data will be captured from all trial participants. Participants will self-report estimates of usual time and resources spent managing incontinence and, within the facility-based procedures, time spent managing incontinence over the 24 h periods assigned to leaking freely, using cup, and using cup+. During community-based follow up, these individuals will be surveyed with the same questions monthly through three months [78, 79]. We will micro-cost fistula-related resources using published prices [80]. Community-administered surveys will include measures of cost for supplies related to fistula management and productivity lost; we will supplement the TM data with data on opportunity costs collected within our acceptability assessment (Aim 2). Clinic treatment cost (e.g., human resources and provider time effort) will be collected from facility managers. In addition, average sales and wholesale prices of supplies will be combined with utilization patterns captured during TM to calculate fistula management cost and to supplement participant-reported costs. The three data sources will comprehensively capture the range of fistula management costs. To minimize discrepancy, we will provide detailed activity categories in our survey (e.g., inserting/removing cup, washing, etc.). To maximize rigor, participants will be trained on 24-hour activity reporting during their time at the facility.

### Data analysis

#### Quantitative

We will assess missingness and normality of variables; multiple imputation (> 5% missingness), transformation and other techniques will be considered. Data will be summarized descriptively. Intention-to-treat will be the primary analysis. Study data management procedures are available upon request by the study team.

#### Effectiveness outcomes

##### Urine leakage

We will estimate the effectiveness of each intervention (cup and cup+) on six-hour urine leakage through linear mixed-effects regression analysis with subject-specific random intercepts. This approach is the superior approach for maximizing statistical power as it uses all collected data to simultaneously model the treatment and period effects (carry-over effects are not anticipated for this trial); allowing us to simultaneously model our three primary comparisons of cup vs. leak freely, cup + vs. leak freely, and cup vs. cup +  [81, 82].

##### Quality of life

We will compare mean difference in quality of life score as measured by the post-fistula repair reintegration instrument [83] at 1–3 months compared to baseline using McNemar’s test by intervention and will separately model the trajectory of quality of life across our five assessment points using mixed-effects linear regression analysis of assessment points within individuals. Comparative effectiveness of the cup vs. cup + on quality of life will be assessed by comparing the respective quality of life change using paired t-test. Perceived leakage will be assessed similarly. As we observed a wide variability in baseline leakage in our feasibility study (63.2 ml ± 49.2) [84] and clinical cut points leakage reduction are not established, we will use quality of life estimates to identify a clinically significant leakage reduction. Quantitative measures of *short-term user acceptability* will be described using appropriate distributional statistics and compared between cup vs. cup + using paired t-test (continuous) or McNemar’s test (categorical). Quantitative measures of *long-term acceptability* will be described by cup vs. cup + through modeling their trajectory across community assessment and comparing these statistically using mixed-effects linear regression analysis. In secondary analyses we will assess various potential confounders will for association with the primary outcome variable (% change in volume of urine leaked at 6 h of use) as well as with user acceptability score. A direct relationship will also be assessed between effectiveness (self-perceived change in volume of urine leaked) recorded at 24 h of use and at 1, 2, and 3-month follow-up for both intervention models.

### Costing

To inform the social impact of fistula management and future cost-effectiveness assessment, we will summarize fistula management cost data descriptively for cup, cup+, and usual non-surgical methods; measurement of central tendency will be generated. We will compare fistula management costs by use domains between cup, cup+, and usual non-surgical methods using chi-square tests, Kruskal-Wallis H tests, and Nemenyi post-hoc tests.

### Qualitative

Interviews will be transcribed and coded to address research questions and open-coded for additional themes [85, 86]. We will use the framework analysis method to provide a systematic model for managing and integrating the quantitative and qualitative data on user acceptability, qualitative data on implementer acceptability, and facilitators and barriers to implementation [87]. We will compare acceptability constructs within and between users and implementers. Strategies for rigor include seeking and incorporating feedback from Community Advisory Board members and using an audit trail [88]. Analysis of qualitative outcomes of acceptability will follow a two-stage systematic process and interviewers who translated/transcribed will help the binational research team with coding. First stage: data coding and classification by reviewing transcripts for conceptual categories. Two types of codes: deductive and emergent. First, deductive codes that represent expected influences will be applied, taken from existing literature and interview guide oriented by the CFIR [75, 76, 89]. This will include examination of acceptability by difference between those recruited from northern or southern regions, different cultural groups, and rural/urban to examine different concerns across settings. For stakeholder interviews recruiting men and women, we will examine acceptability differences by gender/sex. Next, emergent codes will represent themes not expected by the researchers. A codebook will include detailed code description, code application criteria, and examples. Data will be analyzed by different dimensions and commonalities of themes, patterns, and linkages, and by participant characteristics. Exploratory analyses by user characteristics (e.g., fistula size, incontinence severity, or physical activity intensity) may help identify optimal users for each of the device modifications that could be examined in future research. Integration of quantitative and qualitative data from quantitative and qualitative findings via joint analysis [62] will also contextualize effectiveness outcomes through our sequential explanatory approach.

### Softwares

Qualitative analyses will be carried out in Nvivo v.11. Joint analyses will be generated in excel or in Nvivo v.11. All quantitative analyses will be carried out in Stata 17.

### Data and safety monitoring

A formal safety review process will be implemented for the duration of the study which will include a data and safety monitoring board of three individuals with expertise in clinical obstetrics, biostatistics, and medical ethics not affiliated with the study. Formal DSMB meetings will occur every six months, and ad-hoc meetings may be convened. At DSMB review, or any other time, the DSMB may recommend that the study proceed as designed, proceed with design modifications, or be discontinued. Study investigators will routinely review participant safety data during their weekly team meetings and will convene on an ad hoc basis to make decisions regarding the handling of any significant safety concerns in conjunction with the DSMB. If necessary, experts external to the investigative team or the DSMB with expertise in the appropriate areas may be invited to join safety review activities.

Interim analyses will be carried out by the study data manager for DSMB review formally halfway through the clinic-based assessment phase data collection, i.e., when 50 study participants have completed the study. Findings from the pilot period will be used to inform potential modifications to data collection or other study procedures. Interim analysis of efficacy data when about half the study events are recorded, using stringent rules for stopping the trial early in case of positive effect. Interim trial data will be kept confidential and restricted to the DSMB, except if early stopping of the trial is advised. In addition to recommendations from the DSMB, the study team will seek the input of the Community Advisory Board to help inform decisions about modifications to improve study rigor and appropriateness, among other aspects.

#### Participant removal

Study physicians will consider a participant’s removal from the study if there is harm caused to participant with use of either cup or cup + that prevents their ability to continue using the devices for the planned duration.

#### Stopping rules

Criteria for stopping for harm or significant concerns about safety (e.g., life-threatening or results in death, hospitalisation or prolongs existing hospitalization, results in disability or incapacity, congenital anomaly or birth defect) or observed treatment effectiveness include: a statistically significant difference (*p* < 0.001) according to the Haybittle–Peto boundary. Serious adverse events (SAEs) will also be monitored continuously during data entry, and they will be reported to the DSMB and IRBs. Preliminary analyses will be done by the study statistician, who is unblinded and presents unblinded interim analyses to the DSMB, according to predetermined stopping rules. Recommendations to stop or alter the design or conduct of the trial will be done by the DSMB in instances of high effectiveness and/or poor adherence or unacceptable side effects that can be attributed to the study intervention following pre-determined stopping rules and criteria. Safety reviews will look at both anticipated and unanticipated adverse events. In case DSMB recommends stopping the trial early, a meeting will be held between the study investigators and the DSMB to decide on the trial discontinuation. In case of disagreement, the advice of independent external experts will be sought. A report will be submitted to Korle Bu Teaching Hospital Ethical Committee, Ghana Food and Drug Administration, Amref Research and Ethics and Scientific Review Committee, and UCSF Human Research Protection Program as soon as possible and not later than 10 working days.

## Discussion

Although insertable devices have been used previously to improve women’s health, [57, 90] a paradigm shift will be required to expand comprehensive fistula care to include therapeutic self-management of fistula-related urine leakage with an insertable device as women await surgery or for whom surgery was unsuccessful. Our planned research directly targets the evidence needed to influence such a shift. We extend current approaches to fistula management in concept and methods employed to help overcome fistula management challenges due to limited global fistula surgical capacity, through technological and conceptual innovation [7, 8]. We have planned robust effectiveness testing of two promising cup models for tertiary prevention of fistula which are likely to improve quality of life through reducing the intensity of incontinence management, thus allowing women to engage longer in their normal activities. Technocratic solutions must prioritize an understanding of both user and implementer preferences; thus, we have incorporated implementation research with an early and sustained focus on acceptability in a novel target population and focus among both users and implementers.

Previous research in lower-resource settings has lacked user input regarding insertable devices, [27, 52, 91, 92] with user-technology interactions confounding impact. More recent research on menstrual cups has prioritized acceptability; [30] however, acceptability of cup use for monthly menstruation may be distinct from acceptability of cup use for daily urinary incontinence management. Furthermore, prior studies of menstrual cup acceptability have lacked a human centered design approach [93] to ideate, test, and integrate stakeholder feedback throughout the research and development process. Our research will maintain this focus on acceptability.

We employ an assets-based approach to prioritize women’s agency to self-manage their leakage. Within a resource-constrained setting where surgical capacity does not meet women’s needs, women are resilient in their attempts to cope but lack access and means to appropriate tools [10, 51, 90]. Our study’s approach utilizes a salutogenic (rather than pathogenic) framing to promote the self-esteem and coping abilities of individuals, [94] which stakeholders agreed was a novel framing, [10] and maintains a focus on rapid translational gains for clinical intervention and useful information for key decision-makers.

This innovation, if effective in this context, could reduce stigma and significantly improve quality of life by reducing fistula leakage [95, 96]. Reduced incontinence after fistula repair leads to significant quality of life improvement, [18–21] likely through both the resolution of physical symptoms and its impact on social, marital, and economic integration and emotional wellbeing. The potential impact of the cup/cup + may be as much related to user characteristics (e.g., anatomy and physiology, beliefs about vaginal devices) as much as contextual factors (e.g., health system characteristics, cultural norms). Where surgical capacity is low, women with fistula may be more vulnerable to stigma as they wait months to years for surgery [25, 97] and could use the cup/cup + while waiting [25]. In cases of surgical failure, women may use the cup/cup + indefinitely, as appropriate. Varied access to repair across lower-resource settings highlights the importance of examining context to plan strategies for the implementation of such tools [90, 98, 99 ]. Implementation science frameworks, like the Consolidated Framework for Implementation Research (CFIR) that has recently been adapted in an attempt to increase compatibility for use in low- and middle-income countries, [75, 76] guide analysis of the diverse factors influencing implementation, such as acceptability, thereby informing selection of strategies to address implementation challenges [100, 101].

If the devices are identified as effective and acceptable for long-term use, we will seek to initiate a multi-site effectiveness-implementation trial in sub-Saharan Africa to evaluate stakeholder-generated implementation strategies. The research activities herein position us to optimally design the next steps of our research program on improving comprehensive fistula care, including the identification of ideal users within a larger trial and measures to inform subsequent cost-effectiveness or cost-benefit analysis, as appropriate. We expect that our work to expand the evidence base on non-surgical management options will ultimately result in a positive tangible impact on the quality of life of women with fistula through limiting time spent managing fistula urine leakage, reducing vulnerability to stigma, and empowering women to engage with confidence in social and economic activities.

### Dissemination plans

The study team plans to communicate trial results to participants where possible through community meetings and to healthcare professionals and researchers via presentation of findings via academic conferences and publication in peer-reviewed, scholarly journals. As part of qualitative data collection, preliminary findings will be shared with fistula stakeholders prior to interviews. Lastly, the community advisory board will be included in data interpretation and will be aware of ongoing analyses throughout the trial. We plan to develop a policy brief to concisely summarize our findings and share this with stakeholders, including policymakers who can use our evidence to shape relevant fistula programming and policy to include implementation of non-surgical options for fistula management in Ghana, Kenya and elsewhere. Study data will be deidentified and made publicly available after conclusion of the trial and all primary analyses.

### Study status

This study was funded by the Eunice Kennedy Shriver National Institute of Child Health and Development in April 2022. We completed our pilot test of our research protocol in October 2022 and enrolled our first study cohort on April 15, 2023. Currently 16 participants have completed the study. Cohorts will be consistently enrolled across both study sites for approximately two years, after which the data analysis, interpretation, and dissemination of study findings will occur. This study is registered with ClinicalTrials.gov (NCT05444504) and the Pan African Clinical Trial Registry (202209466217416).

## Data Availability

No datasets were generated or analysed during the current study.

## Abbreviations

CFIR: Consolidated Framework for Implementation Research
IQR: Interquartile range
NICHD: National Institute of Child Health and Human Development
OF: Obstetric fistula
TFA: Theoretical Framework of Acceptability
UNFPA: The United Nations’ Population Fund
VVF: Vesicovaginal fistula
